# An open access pilot freely sharing cancer genomic data from participants in Texas

**DOI:** 10.1038/sdata.2016.10

**Published:** 2016-02-16

**Authors:** Lauren B. Becnel, Stacey Pereira, Jennifer A. Drummond, Marie-Claude Gingras, Kyle R. Covington, Christie L. Kovar, Harsha Vardhan Doddapaneni, Jianhong Hu, Donna Muzny, Amy L. McGuire, David A. Wheeler, Richard A. Gibbs

**Affiliations:** 1 Dan L. Duncan Cancer Center, Baylor College of Medicine, Houston, Texas 77030, USA; 2 Center for Medical Ethics and Health Policy, Baylor College of Medicine, Houston, Texas 77030, USA; 3 Human Genome Sequencing Center, Baylor College of Medicine, Houston, Texas 77030, USA

**Keywords:** Genetics research, Cancer genomics, DNA sequencing, Medical genomics

## Abstract

Genomic data sharing in cancer has been restricted to aggregate or controlled-access initiatives to protect the privacy of research participants. By limiting access to these data, it has been argued that the autonomy of individuals who decide to participate in data sharing efforts has been superseded and the utility of the data as research and educational tools reduced. In a pilot Open Access (OA) project from the CPRIT-funded Texas Cancer Research Biobank, many Texas cancer patients were willing to openly share genomic data from tumor and normal matched pair specimens. For the first time, genetic data from 7 human cancer cases with matched normal are freely available without requirement for data use agreements nor any major restriction except that end users cannot attempt to re-identify the participants (http://txcrb.org/open.html).

## Background & Summary

New sequencing, data analysis, and visualization methodologies must be developed to realize the full potential of cancer genomic medicine as part of precision medicine^1^. Limited availability of accessible genomic reference datasets is a major bottleneck to maximizing this potential in a way that engages researchers and the public. It also hampers education in modern cancer bioinformatic analysis techniques using real world reference datasets. Most individual-level genomic data is currently accessible only through controlled-access initiatives (e.g., dbGaP, the database for genotypes and phenotypes; or cgHub, the cancer genomics hub), which require security and data-use agreements. Restricting access to individual-level genomic data protects against the risk of unauthorized re-identification, thereby reducing the possibility of discrimination against research participants and their families. However, patient identification is still possible in a controlled access database, whether intentional or not, and the broad policy of restricting access to these data inhibits research, as data in controlled access databases are not used as often as data in open access databases^2^. In addition, data use agreements not only limit who can access the data, placing a barrier between interested patients and genomic researchers^3^, but also where the data can be analysed. The latter hampers use of highly-accessible cloud-based platforms that have appropriate data control, access and accountability structures^4^.

Much of the ethical debate about data sharing has focused on the identifiability of genomic data and balancing privacy risks with the benefits of making data broadly available to the research community^5–8^. There is an ethical responsibility to ensure not only that participants’ privacy is protected, but also that the utility of the data that they contribute for research is maximized^2^. In fact, limiting access to genomic data from participants who wish to share their data openly undermines their autonomy and may be viewed as unjustifiably paternalistic. We have previously argued that participants should be informed of both the risks and benefits of different levels of data sharing (i.e., controlled versus open access) and be allowed to make decisions about how broadly they are comfortable sharing their data^8,9^. A remaining concern lies in the notion that research participants may not be able to provide truly informed consent for genomic data sharing. Research participants often have poor recall of information presented in informed consent documents^10–12^ or with whom they had agreed to share their genomic data^13^. Additionally, obtaining consent from cancer patients in particular may be challenging due to protracted periods of psychological and physical stress associated with diagnosis and care^14^.

**Figure 1:**
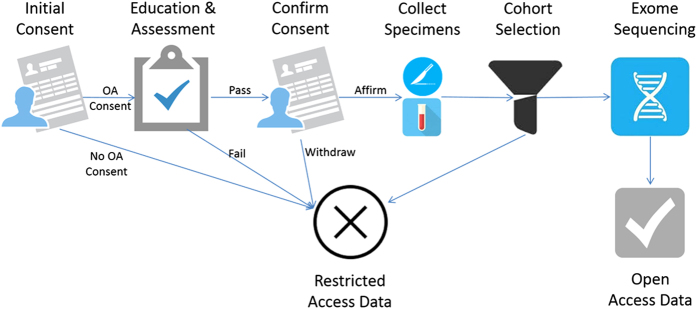
The Texas Cancer Research Biobank (TCRB) Open Access (OA) data sharing workflow. Participants provided initial consent to participate in OA data sharing. Those who granted consent were educated on risks and benefits then quizzed to test understanding and asked whether they wanted to opt out of OA sharing. Tumor and normal blood specimens from those who showed comprehension and reaffirmed consent were then obtained and subjected to whole exome sequencing.

Here, we describe an OA project and its resulting genomic dataset demonstrating that many cancer patients could and would provide true informed consent (Fig. 1). Of these participants, seven were selected for OA release. Only a subset was released to protect privacy to the fullest extent possible. The TCRB provided a model for value-based collections^15^ in which 1) the highest possible data and specimen quality were maintained through standard consents, specimen and data collection protocols, pathological review procedures, material transfer agreements, and other control and documentation mechanisms that are freely available^16^; and 2) society can benefit through international sharing of genomic data through this OA effort and through TCRB’s contributions to the ICGC, the International Cancer Genome Consortium, and TCGA, the Cancer Genome Atlas^17,18^. As sequencing and data analytic technologies improve, the definition of quality must be broadened to tackle the next phase of cancer genomic research. TCGA accepted only the ‘ideal’ of frozen primary tumours with matched normal samples from participants who had not had prior treatment and whose samples met other stringent quality factors. A new frontier in cancer genomics research will be utilization of ‘real world’ cases—longitudinal studies of smaller specimens such as biopsies; multiple biopsies of a single tumor to better model heterogeneity; and evaluation of tumours from patients who have had prior antineoplastic interventions or whose samples do not meet TCGA’s rigorous pathological quality control standards. Some such specimens are included in this dataset to present an opportunity for the community to develop methods to better utilize data from these types of cases, in an attempt to maximize benefit to cancer patients, many of whose samples will never meet ‘ideal’ conditions for genetic research.

Efforts such as the Personal Genome Project in the US, Canada, UK and Austria are publicly sharing genomic and clinical data from healthy participants^19^. To our knowledge, the TCRB OA dataset is the first individual-level, open-access genomic data release targeted to human cancers. Lowering barriers to access increases potential for misuse of the data, but with appropriate informed consent and reasonable steps taken to protect patient confidentiality, this dataset confirms that many patients recognize that positive future benefits can outweigh these risks. This consent coupled with public access to a large pool of readily-available data is critical to addressing particularly difficult challenges with human cancers.

## Methods

### Obtaining informed consent

All work was carried out as part of an IRB-approved protocol (BCM IRB H-32711), which utilized a main consent document for general participation in TCRB with an opt-in consent addendum for OA data release. Of 194 TCRB participants offered the option of signing the opt-in addendum participating in OA sharing out of >2,500 total participants, more than half agreed to open access data sharing at time of consent. Annotated TCRB specimen and data collection consent and the OA opt in consent documents are available at http://txcrb.org/resources.html. To address concerns about whether patients can provide truly informed consent regarding the potential risks of genomic data sharing, a subset of the OA participants (*n*=37) were educated on risks and societal benefits of data sharing. The educational materials are available at http://txcrb.org/privacy.html. Participants were surveyed to assess their comprehension, risk tolerance, and subjective comfort with OA data release. Each participant was again queried, post-survey, to reconfirm their choice to take part in the OA data sharing option. The majority demonstrated adequate understanding of the possible privacy and discrimination risks, yet still elected to allow their data to be openly shared. The work described in Pereira *et al.* ^9^ is one clear example that many, though not all, cancer patients indeed desire to participate in activities that could have broad-reaching, positive impacts to public health for reducing cancer mortality and morbidity, and have the capability to make an informed choice.

### Selecting the OA cohort

Approximately 20% of the 37 participants (*n*=7) who were surveyed and still consented to OA data sharing were selected for inclusion in the TCRB cancer OA dataset. To further reduce risk of reidentification, none of the OA participants had rare ethnicities or tumor types as defined by SEER (Surveillance, Epidemiology, and End Results program) statistics. Figure 1 shows the process for this and subsequent steps in creation of this dataset.

### Tumor and normal collection

Blood was collected from participants in PAXgene Blood DNA tubes, and DNA was isolated using the PAXgene Blood DNA kit (PreAnalytiX, Qiagen, Valencia, CA). The tumor pancreas tissue specimens were collected shortly after resection and stored in a protease inhibitor solution (Roche Applied Science, Indianapolis, IN), RNAlater (Qiagen), or snap frozen, and stored at −80C. The blood sample specimen was used as matching normal control. DNA was isolated from 50–100 mg tissue fragments using the GentraPuregene kit (Qiagen). The quality of the DNA samples were ascertained by electrophoresis and determined to be of high quality (size >23 kb) with no visible degradation in blood or tumor samples.

To implement genomic sequencing approaches in ‘real world’ specimens, it is imperative to detect variants in clinical samples that have reduced tumour cellularity, e.g., as a consequence of neoadjuvant or other prior therapy. We devised methodologies to overcome the challenges associated with extensive desmoplastic stroma that is characteristic of the majority of pancreatic tumours, and these strategies facilitated the discovery of novel molecular mechanisms in the pathophysiology of this disease. The cellularity of each primary sample was estimated through pathological review, deep amplicon-based sequencing of exons 2 and 3 of *KRAS* (average depth of 1,000×), and single nucleotide polymorphism (SNP) array-based cellularity estimates using a novel algorithm qpure^20^. Clinical and pathological annotations for each case are shown in Table 1.

### Whole exome sequencing

#### Library preparation

DNA samples were constructed into Illumina paired-end pre-capture libraries according to the manufacturer’s protocol (Illumina Multiplexing_SamplePrep_Guide_1005361_D) with modifications as described in the BCM-HGSC Illumina Barcoded Paired-End Capture Library Preparation protocol. Libraries were prepared using Beckman robotic NXp and FXp model workstations. The complete protocol and oligonucleotide sequences are accessible from the HGSC website (https://hgsc.bcm.edu/sites/default/files/documents/Illumina_Barcoded_Paired-End_Capture_Library_Preparation.pdf). Briefly, 1 ug of DNA in 100 ul volume was sheared into fragments of approximately 300–400 base pairs in a Covaris plate with E210 system (Covaris, Inc. Woburn, MA) followed by end-repair, A-tailing and ligation of the Illumina multiplexing PE adaptors. Pre-capture Ligation Mediated-PCR (LM-PCR) was performed for 7 cycles of amplification using the Phusion High-Fidelity PCR Master Mix (NEB, Cat. no. M0531L). Universal primer IMUX-P1.0 and a pre-capture barcoded primer IBC were used in the PCR amplification. In total, a set of 12 such barcoded primers were used on these samples. Purification was performed with Agencourt AMPure XP beads after enzymatic reactions. Following the final XP beads purification, quantification and size distribution of the pre-capture LM-PCR product was determined using the LabChip GX electrophoresis system (PerkinElmer).

#### Exome capture

For exome capture, four pre-capture libraries were pooled together (~300 ng/sample, 1.2 ug/pool) and hybridized in solution using the VCRome 2.1 Design^21^ supplied by NimbleGen according to the manufacturer’s protocol *NimbleGen SeqCap EZ Exome Library SR User’s Guide (Version 2.2)* with minor revisions. Human COT1 DNA and full-length Illumina adaptor-specific blocking oligonucleotides were added into the hybridization to block repetitive genomic sequences and the adaptor sequences. Post-capture LM-PCR amplification was performed using the Phusion High-Fidelity PCR Master Mix with 14 cycles of amplification. After the final AMPure XP bead purification, quantity and size of the capture library was analyzed using the Agilent Bioanalyzer 2100 DNA Chip 7500. The efficiency of the capture was evaluated by performing a qPCR-based quality check on the four standard NimbleGen internal controls. Successful enrichment of the capture libraries was estimated to range from a 6 to 9 of ΔCt value over the non-enriched samples.

#### Sequencing

Library templates were prepared for sequencing using Illumina’s cBot cluster generation system with TruSeq PE Cluster Generation Kits (Cat. no. PE-401–3001). Briefly, these libraries were denatured with sodium hydroxide and diluted to 6–9 pM in hybridization buffer in order to achieve a load density of ~800 K clusters/mm^2^. Sequencing runs were performed in paired-end mode using the Illumina HiSeq 2000 platform. Each library pool was loaded in a single lane of a HiSeq 2000 flow cell, and each lane was spiked with 2% phiX control library for run quality control. The sample libraries then underwent bridge amplification to form clonal clusters, followed by hybridization with the sequencing primer. Using the TruSeq SBS Kits (Cat. no. FC-401–3001), sequencing-by-synthesis reactions were extended for 101 cycles from each end, with an additional 7 cycles for the index read. Sequencing runs generated approximately 300–400 million successful reads on each lane of a flow cell, yielding 7–13 Gb per sample. For exome sequencing yields, samples achieved an average of depth of coverage of 200X over exonic regions.

### Whole genome sequencing

#### Library preparation

For most cases, there was not sufficient quantities of biospecimen available to perform whole genome sequencing (WGS). For these cases, only WEX data exist. For two cases, however, it was possible to perform WGS. Library templates were prepared for sequencing using Illumina’s cBot cluster generation system with TruSeq PE Cluster Generation Kits (Cat. no. PE-401–3001). DNA (0.5 ug) in 70 ul volume was sheared into fragments of approximately 500–700 base pairs using Covaris S2 system (Covaris, Inc. Woburn, MA). Briefly, these libraries were denatured with sodium hydroxide and diluted to 6–9 pM in hybridization buffer in order to achieve a load density of ~800 K clusters/mm^2^. Illumina multiplexing PE adaptors with barcode sequences were added to the sample at the time of ligation. Pre-capture Ligation Mediated-PCR (LM-PCR) was performed for 6–8 cycles using the Library Amplification Readymix containing Kapa HiFi DNA Polymerase (Kapa Biosystems, Inc., Cat # KK2612) and universal primer-pair IMUX-P1.0 and IMUX-P3.0. 4) For purification of the fragmented DNA, 0.8X AMPure XP (Beckman, Cat. No. A63882) was used as opposed to using 1.8X for preparation of WES libraries.

#### Sequencing

Tumor libraries were sequenced in four lanes, and normal libraries were sequenced in two lanes of a HiSeq 2000 flow cell, yielding approximately 60X and 30X coverage respectively. Each lane was spiked with 2% phiX control library for run quality control. The sample libraries then underwent bridge amplification to form clonal clusters, followed by hybridization with the sequencing primer. Sequencing runs were performed in paired-end mode using the Illumina HiSeq 2000 platform. Using the TruSeq SBS Kits (Cat. no. FC-401–3001), sequencing-by-synthesis reactions were extended for 101 cycles from each end, with an additional 7 cycles for the index read. Sequencing runs generated approximately 300–400 million successful reads on each lane of a flow cell, yielding ~11 Gb per sample.

### Read mapping and variant calling

Binary bcl files output from the HiSeq 2000 were processed with BclConvertor 1.7.1 software available from Illumina. All reads from the prepared libraries that passed the Illumina Chastity filter were formatted into FASTQ files, which were aligned to the human reference genome build 19 version GRCh37-lite using BWA 0.5.9rcl (Burrows-Wheeler Aligner)^22^. Default parameters were used for alignment, except for a 40 bp seed sequence with 2 mismatches in the seed and a total of 3 mismatches overall allowed. Subsequent base quality recalibration and local realignment around known indel sites was performed by GATK 3.3 (ref. 23). Variant calling was performed using on samples using Atlas-SNP2 (ref. 24), Atlas-Indel2 (ref. 25), and PInDel^26^. Variants from tumor and matched normal pair samples were compared with each other and with NCBI human reference genome version 19 (hg19) to generate primary calls, which then were annotated using Annovar^27^, COSMIC^28^, and dbSNP^29^ and split into germline or somatic calls in VCF files. The germline and somatic calls were filtered by curators for quality and are available in MAF files. FASTQ, BAM, VCF and filtered MAF and available clinical annotations are freely available on the TCRB website (Data Citation 1) and FASTA and BAMs also within SRA, the Sequence Read Archive (Data Citations 2 and 3).

### Code availability

All software used to generate the sequence data and to manage biospecimen and clinical annotations is freely available. Specific software versions and references for code are provided as in-line references above.

## Data Records

FASTQ reads and BAM data records for tumor (T) and normal (N) specimens from each case are freely available along with their conditions of use are freely available on the Texas Cancer Research Biobank website, http://txcrb.org/open.html (Data Citation 1). Clinical annotations available for these cases are defined in Table 1. Other than a click-through agreement to acknowledge the conditions of use, requirement to create an access account for auditing purposes, and include these conditions within any re-sharing of the data, there are no additional barriers to data access on this portal. User accounts are valid for 30 days and can be renewed. All or some of these data may be downloaded, shared and redistributed for research and educational purposes in accordance with their conditions of use.

To ensure sustainable availability of the data, they are also deposited within SRA. We created the Texas Cancer Research Biobank Open Access Data Sharing Umbrella Project (Accession: PRJNA285925) under which two platform-specific projects were created—the subproject entitled the Texas Cancer Research Biobank Open Access Data Sharing: Exome Project (Data Citation 2) that includes all seven cases and the subproject entitled the Texas Cancer Research Biobank Open Access Data Sharing: Genome Project (Data Citation 3), for which sufficient genetic material remained for whole genome sequencing after whole exome sequencing was performed and includes cases 6 and 7.

The NCI and others are considering mechanisms to bring the computational capabilities to the data to circumvent issues with transferring large files over networks. For end users who lack sufficient local computation and/or storage capabilities or for whom data downloads may be challenging, a third copy of the data records, their conditions of use, and the HGSC’s Mercury informatics pipeline is available on the DNAnexus cloud. The click-through conditions of use and data can be accessed at https://dnanexus.github.io/tcrb-data/.

Additional data records such as whole genome sequences may be generated from these cases, they will be added to each of these repositories.

## Technical Validation

The TCRB utilized a secure, database-backed web application called Acquire^30^ (code available at https://github.com/BCM-DLDCC/Acquire) for tracking specimens and their annotations. Through its modules, it supports the full lifecycle of biobanking operations, from collections to quality control testing. Public researchers can use the specimen request module to electronically search for and request available specimens. Acquire greatly facilitated non-OA TCRB donations to the TCGA and ICGC.

As TCRB tissue advocates at sites across the state of Texas consented patients, collected specimens, and entered data into Acquire. The system automatically assigns a barcode and UUID (universally unique identifier) to each specimen, aliquot and derivative. These identifiers are completely masked and contain no PHI or other data that can be mapped back to participants, such that the system’s administrators held the mapping for the UUIDs to participant identifiers acted as the TCRB honest broker. All specimens underwent initial review by expert pathologists for disease diagnosis at the Texas hospital or clinic at which the patients were consented. The TCRB’s own expert pathologists reviewed slides cut from the tumor specimens to validate the initial diagnosis and other pathological characteristics such as percent cellularity and necrosis according to TCRB pathology procedures. When minimal data entry was complete, tumor and normal matched pairs were added to shipment reports in Acquire and the data were transmitted to HGSC’s laboratory information management system (LIMS). These reports use only masked specimen identifiers from Acquire. A copy of the shipment report was also printed and included with the specimens to provide a shipping manifest as a secondary identification measure. Specimen barcodes were scanned to confirm the identity of the tumor and normal samples and cross-referenced against the list of expected barcodes. Verified specimens were given a de-identified HGSC accession number for storage in their LIMS. HGSC personnel isolated nucleic acids and obtained quality measurements such as total nucleic acid concentration, RIN values for RNA or polyacrylamide gels for DNA. These metrics were saved to HGSC’s LIMS and also entered into Acquire as annotations on the specimen. Consent and specimen collection forms as well as procedures for specimen collection and shipping are available at www.txcrb.org/resources.html.

Research coordinators re-examined the medical records of each OA participant relative to the data entered into Acquire to assure that clinical annotations were correct. The Acquire coordinator compiled clinical and pathological annotations with HGSC accession numbers and other data for each case.

To verify that the resultant BAM files were not corrupted and could be re-processed from FASTQ files in another environment, the BCM files were re-processed using DNAnexus cloud-based instance of the HGSC’s Mercury pipeline^31^, pertinent algorithms in which are described within the Methods section. All passed this edit check.

**Figure 2:**
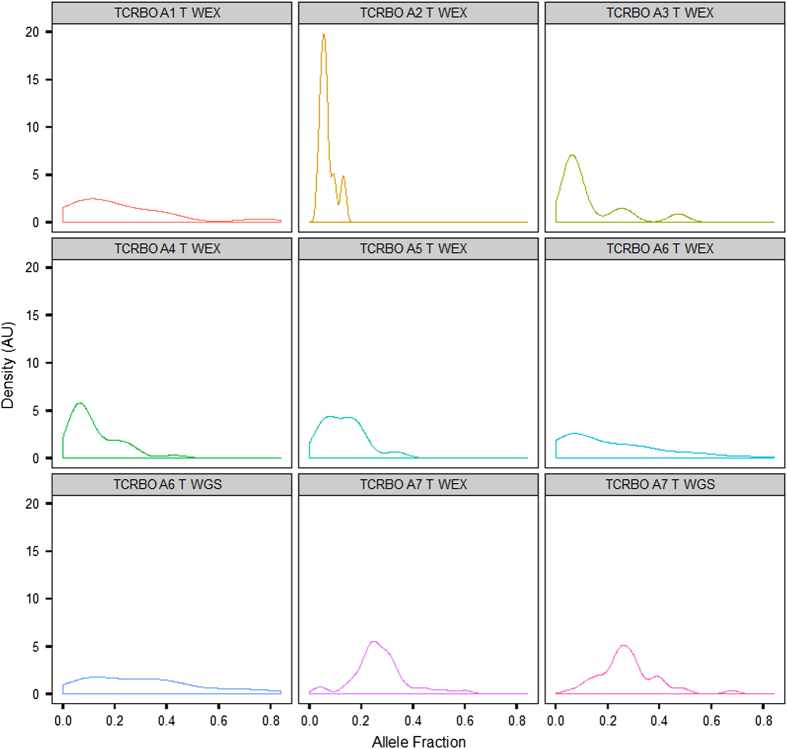
Allele fraction density plot for all TCRB-OA samples. Variants were restricted to the coding region so that WEX and WGS samples are comparable. Allele fraction (ratio of variant reads to total reads) density is plotted for each sample.

Quality analyses were performed on WEX and WGS data, and all data files fall within acceptable parameters as shown in Table 2. All samples were of adequate quality for variant analysis as determined by concordance and contamination estimates based on SNP array (see Table 2). We also compared the ratio of novel variants to known high-quality dbSNP variants detected from our somatic variant analysis and found that all samples were within acceptable limits, no abundance of population variation was noted in the somatic calls indicating that the samples are properly paired and show no detectable contamination from other samples above background. Allele fraction analysis, comparing the ratio of variant reads to total reads, of the somatic variants indicated that most of the samples were of low tumor cellularity, generally less than 20% (Fig. 2) with the exception of TCRBOA7 which has an estimated allele fraction peak of 25% and an estimated tumor cellularity of 50%. These features make this cohort quite useful for algorithm developers seeking to tune their callers to low tumor cellularity samples. Table 3 summarizes the SNP and indel counts, driver mutations uncovered in each case and a comparison of the percent cellularity as measured by driver variant allelic fraction and by expert pathologists. In all cases where data were available, pathological cellularity determinations were higher than those by allelic fraction.

## Usage Notes

By downloading or utilizing any part of this dataset, end users must agree to the following conditions of use:

- No attempt to identify any specific individual represented by these data or any derivatives of these data will be made.
- No attempt will be made to compare and/or link this public data set or derivatives in part or in whole to private health information.
- These data in part or in whole may be freely downloaded, used in analyses and repackaged in databases.
- Redistribution of any part of these data or any material derived from the data will include a copy of this notice.
- The data are intended for use as learning and/or research tools only.
- This data set is not intended for direct profit of anyone who receives it and may not be resold.
- Users are free to use the data in scientific publications if the providers of the data (Texas Cancer Research Biobank and Baylor College of Medicine Human Genome Sequencing Center) are properly acknowledged.

Implementation of common vocabularies facilitates semantic interoperability and data reuse. Annotations for OA cases include controlled terminologies for race, gender and ethnicity from the NIH; pathological diagnosis from World Health Organisation’s ICD-O-3 (International Classification of Diseases-Oncology version 3); and tumor stage and grade data from Union for International Cancer Control (UICC/AJCC). Use of standard metadata facilitates syntactic interoperability. The TCRB used standard data elements, file formats and metadata. Because OA data can be downloaded and re-shared on the provision that the conditions of use are included and abided by, all annotations were also included as comments in BAM file headers with a reference to the conditions of use. These metadata were included to ensure that the clinical and pathological data cannot become decoupled from the sequence data.

## Additional Information

**How to cite this article:** Becnel, L. B. *et al.* An open access pilot freely sharing cancer genomic data from participants in Texas. *Sci. Data* 3:160010 doi: 10.1038/sdata.2016.10 (2016).

## Supplementary Material



## Figures and Tables

**Table 1:** TCRB open access clinical and pathological annotations by case.

**Case**	Age*	**Race**	**Gender**	**Ethnicity**	**TX**†	**ICD-O-3 Anatomic Site**	**ICD-O-3 Morphology**	**Tumor % Cellularity**	**pT**	**pN**	**pM**	**Grade**
TCRBOA1	51–60	White	Male	Not Hispanic or Latino	N	Blood	Normal					
						Head of pancreas	8500/3: infiltrating duct adenocarcinoma	10	3	1	0	II
TCRBOA2	61–70	White	Female	Not Hispanic or Latino	Y	Blood	Normal					
						Head of pancreas	8500/3: infiltrating duct adenocarcinoma	60	3	1	0	II
TCRBOA3	51–60	White	Male	Not Hispanic or Latino	Y	Blood	Normal					
						Head of pancreas	8500/3: infiltrating duct adenocarcinoma	20	3	1	0	II
TCRBOA4	41–50	White	Female	Hispanic or Latino	N	Blood	Normal					
						Tail of pancreas	8246/3: neuroendocrine carcinoma, NOS	20	2	1	0	II
TCRBOA5	51–60	White	Male	Not Hispanic or Latino	N	Blood	Normal					
						Head of pancreas	8500/3: infiltrating duct adenocarcinoma	5	3	1	X	II
TCRBOA6	61–70	White	Female	Not Hispanic or Latino	N	Blood	Normal					
						pancreas	8246/3: neuroendocrine carcinoma, NOS	80	3	0	0	low, IIA
TCRBOA7	61–70	White	Male	Not Hispanic or Latino	N	Blood	Normal					
						Lymph node of groin	9690/3: Follicular lymphoma	90	N/A	N/A	N/A	I-II

*Age binned by decade.

†Prior treatment (Yes, Y, or No, N) as defined by TCGA criteria.

**Table 2:** Quality control data for whole exome (WEX) and whole genome (WGS) sequencing strategies by case.

**Case**	**Sample Source**	Sequencing Strategy*	**Total Reads**	**Q30 bases (%)**†	**Median Read Length**	**Mode Read Length**	**Duplicate Reads (%)**	**Average Coverage**	**Bases with 20+ Coverage (%)**	**Contamin-ation**
TCRBOA1	blood	WEX	98,204,556	82.3	207	167	4.34	95	94.6	2.46
	tumor	WEX	103,758,450	82.7	204	161	4.51	99	94.34	2.49
TCRBOA2	blood	WEX	112,293,884	84.9	205	163	6.56	113	95.26	3.01
	tumor	WEX	122,780,752	84.9	211	165	6.19	119	95.57	3.08
TCRBOA3	blood	WEX	116,854,764	89.7	253	200	4.97	119	96.5	2.93
	tumor	WEX	103,017,862	89.9	247	199	4.5	108	96.02	2.94
TCRBOA4	blood	WEX	88,085,950	89.3	211	161	7.61	87	93.94	2.73
	tumor	WEX	99,749,920	90.4	211	161	7.04	107	94.81	2.52
TCRBOA5	blood	WEX	94,774,390	90.0	209	161	7.78	101	94.69	2.56
	tumor	WEX	97,275,566	89.8	212	165	7.71	101	94.87	2.54
TCRBOA6	blood	WEX	122,317,962	92.0	154	131	6.11	158	92.27	2.58
	tumor	WEX	116,876,622	91.9	154	132	5.96	151	93.31	2.52
TCRBOA7	blood	WEX	124,540,044	92.0	198	148	5.41	75	93.24	0.20
	tumor	WEX	128,584,540	92.0	193	147	5.44	77	94.01	0.22
TCRBOA6	blood	WGS	1,133,175,530	81.0	439	397	—	—	—	0.37
	tumor	WGS	2,162,928,452	84.3	435	400	—	—	—	0.26
TCRBOA7	blood	WGS	1,432,195,452	89.8	427	388	—	—	—	0.12
	tumor	WGS	1,524,291,878	88.6	425	387	—	—	—	0.18

*Whole exome sequencing, WEX. Whole genome sequencing, WGS.

†Phred base-calling score, Q, of 30 or above.

**Table 3:** Summary of filtered somatic variants from MAF files of whole exome (WEX) and whole genome (WGS) data by case.

**Case**	Sequencing Strategy*	**Exonic**	**Frame Shift**	**In Frame**	**Mis-sense**	**Non-sense**	**RNA**	**Silent**	**Splice Site**	**Grand Total**	**Driver genes identified**	**Driver Variant Allelic Fraction Cellularity % (Path Cellularity %)**
TCRBOA1†	WEX	—	—	—	5	—	—	5	—	10	KRAS	5 (10)
TCRBOA2† ^,*^	WEX	—	—	—	3	—	—	3	1	7	CDKN2C	8 (60)
TCRBOA3† ^,*^	WEX	—	—	—	8	—	—	2	—	10	KRAS, TP53	from 9–15 (20)
TCRBOA4‡	WEX	—	—	—	9	1	—	12	—	22	—	N.D. (20)
TCRBOA5†	WEX	—	—	1	5	1	—	4	—	11	—	N.D. (5)
TCRBOA6‡	WEX	—	3	3	27	1	—	9	1	44	MEN1, ATXN1	55 (80)
TCRBOA7§	WEX	—	1	—	20	3	—	11	2	37	CREBBP, MLL2, NFE2L3	>50 (90)
TCRBOA6‡	WGS	2	3	2	23	1	2	8	—	41	MEN1	63 (80)
TCRBOA7§	WGS	—	1	5	20	2	2	14	3	47	CREBBP, NFE2L3	>50 (90)

*Whole exome sequencing, WEX. Whole genome sequencing, WGS.

†Pancreatic Infiltrating ductal carcinoma.

‡Pancreatic neuroendocrine tumor.

§Follicular lymphoma.
